# Advances in Research on Bioactivity, Toxicity, Metabolism, and Pharmacokinetics of Usnic Acid In Vitro and In Vivo

**DOI:** 10.3390/molecules27217469

**Published:** 2022-11-02

**Authors:** Hanxue Wang, Min Xuan, Cheng Huang, Changhong Wang

**Affiliations:** 1 Shanghai TCM-Integrated Hospital, Shanghai University of Traditional Chinese Medicine, 230 Baoding Road, Shanghai 200082, China; 2 School of Pharmacy, Shanghai University of Traditional Chinese Medicine, 1200 Cailun Road, Shanghai 201203, China; 3 The MOE Key Laboratory for Standardization of Chinese Medicines and The SATCM Key Laboratory for New Resources and Quality Evaluation of Chinese Medicine, Shanghai Key Laboratory for TCM Complex Prescription, Institute of Chinese Materia Medica, Shanghai University of Traditional Chinese Medicine, 1200 Cailun Road, Shanghai 201203, China; 4 Department of Pharmacy, Qingdao Eighth People’s Hospital, 84 Fengshan Road, Qingdao 266121, China

**Keywords:** usnic acid, lichen, biological activity, hepatotoxicity, metabolism

## Abstract

Lichens are among the most widely distributed plants on earth and have the longest growth cycle. Usnic acid is an abundant characteristic secondary metabolite of lichens and the earliest lichen compound used commercially. It has diverse pharmacological activities, such as anti-inflammatory, antibacterial, antiviral, anticancer, antioxidant, and photoprotective effects, and promotes wound healing. It is widely used in dietary supplements, daily chemical products (fodder, dyes, food, perfumery, and cosmetics), and medicine. However, some studies have found that usnic acid can cause allergic dermatitis and drug-induced liver injury. In this paper, the bioactivity, toxicity, in vivo and in vitro metabolism, and pharmacokinetics of usnic acid were summarized. The aims were to develop and utilize usnic acid and provide reference for its future research.

## 1. Introduction

Lichens, as the most fascinating organisms on earth, are among the most widely distributed plants and have the longest growth cycle, growing throughout the northern temperate zones, especially the subarctic and coastal rainforests of Europe, Asia, and North America [1,2]. Over 600 species of the genus *Usnea* have been discovered worldwide and nearly 90 species have been found in China, widely distributed in the primary forests of many provinces, such as Heilongjiang, Jilin, Shanxi, Gansu, Sichuan, Yunnan, Guangxi, Guizhou, Zhejiang, Fujian, Taiwan, Inner Mongolia, Xinjiang, and Tibet [3,4,5]. As a valuable plant resource, plants from the genus *Usnea* have been widely used in fodder, dyes, food, perfumery, cosmetics, pharmaceuticals, preservatives, deodorants, ecological applications, and miscellaneous purposes throughout the world, particularly in Europe and East Asian countries, such as China, Japan, and India [4,5,6,7,8].

**Figure 1:**
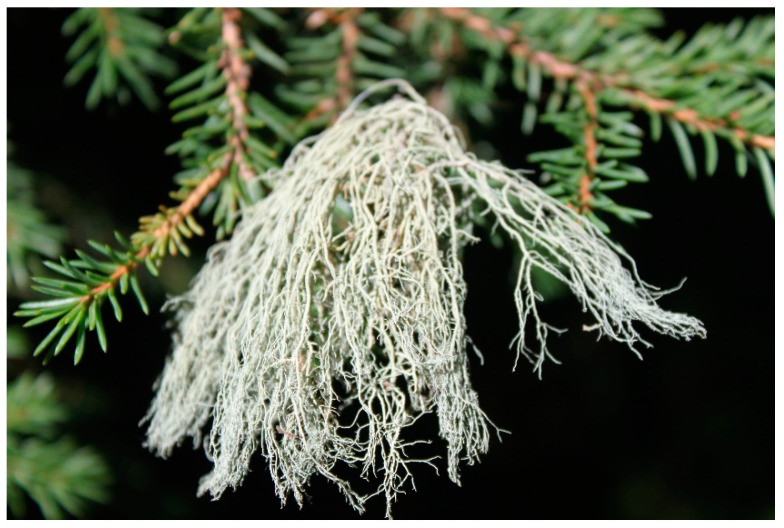
Photograph of *Usnea longissima* taken at Kanas, Xinjiang, China.

Usnea (Figure 1) is the filament plant of *Usnea diffracta* Vain and *Usnea longissima* Ach in the Usneaceae family [9] and has been well recorded in many monographs of medicine, such as the Shennong’s Herbal Classic of Materia Medica and Compendium of Materia Medica. As a commonly used traditional Chinese, Mongolian, Tibetan, and Uighur medicine, *Usnea* was documented in the Drug Standard of the Ministry of Health of People’s Republic of China: Uighur Medicine Fascicule and Chinese Materia Medica: Mongolian Medicine Volume [10,11]. *Usnea* has versatile functions, such as clearing heat and detoxifying, dispelling phlegm, relieving cough, regulating homeostasis and menstruation, and repelling insects and is, thus, used to treat malaria, cough, gasp, tuberculosis, headache, carbuncle, scrofula, acute mastitis, scalds, venomous snake bite, rheumatism, bruises, traumatic bleeding, and irregular menstruation [8,12,13,14,15,16,17,18,19,20,21,22].

**Figure 2:**
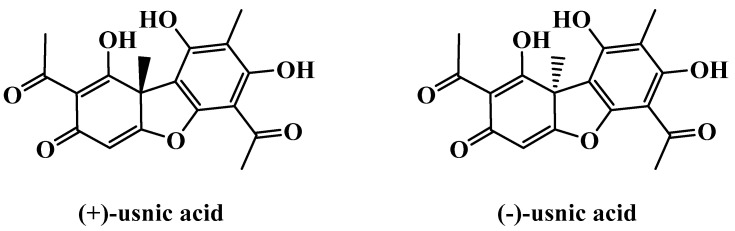
Chemical structures of (+/−)-usnic acid.

Modern pharmacological studies have confirmed that the many secondary metabolites of *Usnea* have various biological activities, specifically antimicrobial activities against *Mycobacterium tuberculosis* and Gram-positive bacteria [16] and antipyretic-analgesic [17], anti-inflammatory [18], antitumor [19,20], and antiviral [21,22] activities; promote wound healing [23,24,25]; exert photoprotection [26,27,28,29]; and induce antioxidative enzymes and alleviate mucosal damage [18,30,31,32]. All these biological activities are related to the chemical components contained in *Usnea*, such as dibenzofuran compounds, including (+/−) usnic acid (Figure 2), longiusnine, and (−)-placodiolic acid, and phenolic acids, including evernic acid, barbatic acid, diffractaic acid, and ramalic acid [4].

Usnic acid is one of the most abundant characteristic secondary metabolites of lichens and the most common and earliest known dibenzofuran derivative, widely existing in the genus *Usnea*, including *U. diffracta* Vain., *U. longissima* Ach., *U. barbata*, *U. antarctica*, *U. rubicunda*, and *U. subfloridana* [9,33]. Since it was first discovered in 1844, usnic acid has become one of the most widely studied lichen metabolites and one of the few commercially available lichen compounds due to its antibacterial, anti-inflammatory, antiviral, and antitumor properties and ability to promote wound healing and other physiological activities [34]. As one of the earliest lichen compounds that initiate commercialization applications, usnic acid is optically active, mostly exists in dextrorotation form in nature, and has been used in dietary supplements and medical drugs. Moreover, it has been added to daily chemical products, such as body wash, perfume, deodorant, and herbicides [35,36,37,38,39].

However, severe hepatic reactions have been reported, including liver necrosis, fulminant hepatitis, and liver failure, in people consuming dietary supplements containing usnic acid [12,13,14,40,41,42,43]. The U.S. FDA received at least 21 case reports of hepatotoxicity after LipoKinetix consumption (includes phenylpropanolamine, usnic acid, 3,5-diiodo-L-thyronine, yohimbine hydrochloride, and caffeine). Hepatoxicity due to the consumption of this supplement has led to one death, one liver transplant case, seven cases of liver failure, ten cases of chemical hepatitis, and four mild hepatic injury cases [44,45]. The U.S. FDA had post warnings about using pure usnic acid or dietary supplements containing usnic acid and ordered the withdrawal of all products containing usnic acid from the market [46]. Drug-induced liver injury (DILI) caused by usnic acid has gradually drawn the attention of medical researchers.

Usnic acid is generally thought to be found only in lichens [35], but some reports claimed that usnic acid is found in the “mushrooms” of kombucha [47]. However, our literature review did not support this claim. In Tibet, China, the local people will pick *Usnea* from trees and make it as tea. They believe that usnic acid alleviates lung disorders. Yunnan snub-nosed monkey, a unique species in China, takes *Usnea* as its main food throughout the year [48,49,50] and *Usnea* accounts for about 50.6–82.1% of the total food intake of the species [51,52,53]. When food is scarce in winter, reindeer and caribou eat litmus lichen, rich in usnic acid, as their main food. They consume usnic acid, which is decomposed by bacteria in their rumina [54,55,56]. Another report indicated that usnic acid may have been associated with the deaths of 400–500 elk in Wyoming in 2004 [57].

Although the numerous biological activities of usnic acid have been elucidated after years of research, the biological activity, toxicity, and metabolism in vitro and in vivo of usnic acid are still the focus of clinical application. In this review, studies of bioactivity, toxicity, and metabolism on usnic acid are explored to provide reference for further research and development and utilization of usnic acid.

## 2. Biological Activity

### 2.1. Anti-Inflammatory Effects

Inflammation is the body’s response to foreign antigens or tissue damage that may result in loss of tissue structure and function.

Different acute doses of usnic acid can reduce swelling in rat foot induced by carrageenan in acute and chronic inflammation rat models and the alleviating effects are dose dependent. However, this phenomenon was significantly reduced only at 100 mg/kg usnic acid [18]. Similar to nonsteroidal anti-inflammatory drugs, usnic acid has an anti-inflammatory mechanism that may inhibit prostaglandin synthesis. Further studies showed that different concentrations of usnic acid and self-microemulsion of usnic acid can reduce tumor necrosis factor-alpha (TNF-α) induced by heat-killed *Escherichia coli* (EC) at varying degrees with a dose–effect relationship [58].

Usnic acid reduces TNF-α level in a dose-dependent manner and inhibits NO production in lipopolysaccharide (LPS)-activated RAW264.7 macrophages [59]. It can inhibit the expression of TNF-α and inducible nitric oxide synthase (iNOS), possibly by inhibiting the nuclear translocation of NF-κB p65 and the degradation of I-κBα and ultimately exerts anti-inflammatory effects. A study [60] explored the anti-inflammatory effects and corresponding mechanisms of usnic acid on RAW264.7 cells stimulated by LPS and found that usnic acid plays an anti-inflammatory role by inhibiting the activation of NF-κB, increasing the production of IL-10 and HO-1, and downregulating the expression of iNOS, IL-6, IL-1β, TNF-α, and COX-2 genes.

Moreover, usnic acid has a neuroprotective effect on Parkinson’s disease (PD), affecting motor dysfunction in a 1-methyl-4-phenyl-1,2,3,6-tetrahydropyridine (MPTP)-induced PD model [61]. The simultaneous administration of MPTP and usnic acid can attenuate MPTP-induced motor dysfunction in a time-dependent manner. Although usnic acid cannot prevent 1-methyl-4-phenyl-pyridine ion (MPP^+^)-induced primary neuronal death by blocking the activation of NF-κB to reduce the activation of astrocytes, usnic acid can inhibit the inflammatory signaling pathway induced by MPP^+^ and protect dopaminergic neurons.

The triketone part in the structure of usnic acid is an important fragment that contributes to its antibacterial, anticancer, and cytotoxic activities. The structure of usnic acid has been modified and the derivatives of usnic acid have been used in evaluating inhibitory activity against tau protein aggregate accumulation and neuroinflammation [62]. These derivatives are synthesized from the reaction of usnic acid with hydrazines and hydrazides. One of the derivatives inhibits the activation of tau-protein-derived hexapeptide AcPHF6 self-fibrillation model and the aggregation of full-length 2N4R tau protein through heparin-induced mechanism. This derivative can reduce NO release in LPS-stimulated mouse microglia BV2 cells at 10 mM and shows low hepatotoxicity, even at 40 mM. Moreover, it enhances cognitive performance in okadaic-acid-induced Alzheimer’s disease (AD) model rats in water maze tests. Thus, it has been considered a novel inhibitor of tau protein aggregate accumulation and neuroinflammation and a potential therapeutic candidate for AD. All animal or cell models and potential mechanisms mentioned in anti-inflammatory activities of usnic acid are listed in Table 1.

The active molecules in natural medicines are the main sources of modern innovative medicines. A large number of naturally active molecules have been reported to have therapeutic effects on inflammatory diseases and many natural products have significant inhibitory effects on inflammation and exert anti-inflammatory effects through a variety of pharmacological mechanisms. Substantial progress has been made in the study of the anti-inflammatory effects of usnic acid, but the mechanism and drug target are still unclear.

### 2.2. Antibacterial and Antiviral Effects

As the main antimicrobial component in *Usnea*, usnic acid has the highest inhibitory effect on Gram-positive bacteria and *Mycobacterium tuberculosis* and exhibits good inhibitory effects on non-tuberculous mycobacteria, *Mycobacterium neoaurum*, *Bacillus cereus*, *Staphylococcus aureus*, *Escherichia coli*, *Pneumococcus*, *Propionibacterium acnes*, and *Enterococcus* [16,58,63,64,65,66,67] but is ineffective against *Pseudomonas aeruginosa* [58]. In addition, the dioxane extract of *Usnea*, which is mainly composed of usnic acid, can significantly inhibit *Bacillus subtilis* and *Pseudomonas fluorescens* [68].

The bacteriostatic activity of usnic acid has been compared with chemically synthesized bacteriostats or preservatives, such as dehydroacetic acid and benzoic acid, and commonly used bacteriostats, such as sorbic acid; the bacteriostatic activity of usnic acid has been found to be comparable to that of sorbic acid but lower than that of dehydroacetic acid [69]. At pH 5–6, usnic acid shows strong inhibitory effects on some common bacteria, molds, and yeast in cream; these effects suggest that usnic acid can be used as a cream preservative, with the advantages of good efficacy and low dosage.

The inhibition mechanism of usnic acid against Gram-positive bacteria has been studied; the antimicrobial activity of usnic acid against methicillin-resistant *Staphylococcus aureus* is due to its ability to destroy bacterial cell membranes [70]. A study demonstrated that usnic acid inhibits bacterial RNA synthesis or disrupts DNA replication in *Bacillus subtilis* and *Staphylococcus aureus* [71]. The antibacterial activity of usnic acid in vitro can be improved through the synergistic effect of verapamil or clarithromycin [72]. Usnic acid has a synergistic effect with gentamicin and an antagonistic effect with levofloxacin [73]. No difference has been observed in combinations of usnic acid and erythromycin, whereas variability has been observed when usnic acid was combined with clindamycin and oxacillin. Hence, usnic acid is a potential therapeutic agent for diseases caused by resistant *Mycobacterium abscessus* strains.

A combination of usnic acid and zinc sulfate has been used as post-surgical adjuvant therapy of human papillomavirus genital infection after radiosurgical treatment. Usnic acid and zinc sulfate adjuvant treatment can improve the time of re-epithelization and reduce recurrences [74]. Another study discovered that usnic acid and other compounds in lichens can inhibit the tumor-promoter-induced Epstein–Barr virus activation [75]. In addition, usnic acid shows weak antiviral activity against Herpes simplex type 1 and Polio type 1 viruses at concentrations of 7.5 and 30 μg per disc, respectively, resulting in over 4 mm of inhibition zone [76]. No activity against HIV has been observed [77].

The antiviral activity of usnic acid and its derivatives against H1N1 influenza virus pdm09 was investigated for the first time in 2012 [22]. Given that the absolute configurations of chiral compounds are often crucial for their biological activities, two isoforms, (+)-usnic acid and (−)-usnic acid and their derivatives, were investigated. For most experimental subjects, (−)-usnic acid showed higher activity, but its biological activity was reversed after usnic acid was chemically modified. The derivatives of usnic acid can be used as anti-influenza compounds, with the prospect of further optimization. However, some results have suggested that usnic acid exerts anti-inflammatory effects by inhibiting NF-κB activation in LPS-stimulated RAW264.7 macrophages [60]. NF-κB plays an important role in stimulating the propagation of influenza virus in cells and its inhibition can greatly reduce the replication activity and infectivity of the virus [78]. Therefore, usnic acid may indirectly reduce virus propagation by inhibiting the cellular proviral pathway.

In a recent study, usnic acid and its salts against SARS-CoV-2 were tested by using in silico methods and in vitro assays [79]. High-throughput virtual screening using a marine natural product database was used in investigating the inhibitors of SARS-CoV-2 protein targets. Considered with molecular properties and compound availability, four natural products, including usnic acid, were analyzed. An immunofluorescence method using a single dose (10 μM) of each compound against SARS-CoV-2 in vitro was used. Three major variants, alpha (UK, B.1.1.7), beta (South Africa, B.1.351), and delta (India, B.1.617.2), were used in evaluating the in vitro inhibition ability of SARS-CoV-2 variants by usnic acid. The SARS-CoV-2 antiviral efficacy of usnic acid is similar to that of remdesivir, the treatment used for emergency patients with COVID-19, approved by the US FDA. The IC_50_ values of usnic acid and remdesivir are 7.99 and 7.42 μM, respectively, and are lower than those of chloroquine and lopinavir. The antiviral efficacy of usnic acid against SARS-CoV-2 varies by variant. The antiviral efficacy of usnic acid against alpha and delta variants is similar to that against the original strain and the IC_50_ of usnic acid against the beta variant is 2.92 μM. In addition, the most likely target protein of SARS-CoV-2 for (+)-usnic acid is Mpro according to MM-GBSA. All bacterial strains, virus and potential mechanisms mentioned in research of antibacterial and antiviral activities of usnic acid are listed in Table 2.

Usnic acid has been actively studied and used due to its wide-spectrum antibacterial activity before the discovery of penicillin. The frequent use of antibiotics has produced drug-resistant bacteria and, thus, whether the discovery and use of natural antibiotics can mitigate this problem deserves further exploration and research.

### 2.3. Antitumor Effects

The antitumor mechanisms of usnic acid can be summarized as the inhibition of tumor cell proliferation, induction of apoptosis, and inhibition of tumor angiogenesis (Table 3).

In vitro studies have investigated the pharmacological activities of six lichen extracts in cancer and inflammation [80]. Usnic acid may be associated with the decreased viability, apoptosis, and cell-cycle inhibition of tumor cells and can induce G1 arrest in human gastric carcinoma cell lines BGC823 [81], human lung carcinoma cell A549 [20], human breast cancer cell line MCF7 and human prostate cancer cell line LNCaP [82], and G2 arrest in human gastric carcinoma cell lines SGC7901 [81]. Moreover, usnic acid can induce S arrest in HCT-116 cells at 100 μM [83]. All these data suggest that usnic acid does not simply induce cell-cycle arrest in all kinds of cells and is involved in different signaling pathways in different cell types. Usnic acid can also inhibit the proliferation of human leukemia cell U937, human osteosarcoma cell MG-63, and human melanoma cell A375 in a time- and dosage-dependent manner [84] and human prostate cancer PC-3M cells in vitro in a concentration-dependent manner [85]. The mechanism of action of usnic acid may be related to the inhibition of DNA replication and RNA transcription in tumor cells and eventually reduces the proliferation rate of prostate cancer cells or accelerates the apoptosis of tumor cells.

A recent study found that usnic acid can inhibit the synthesis of PD-L1 protein by synergistically reducing STAT3 and RAS pathways, reduce PD-L1 expression in HeLa cells, enhance the cytotoxicity of co-cultured T cells to tumor cells, and inhibit the proliferation of cervical cancer cells [86]. In addition, usnic acid can downregulate the expression of PD-L1 by inhibiting cell proliferation, angiogenesis, migration, and invasion, ultimately inhibiting tumor growth.

Usnic acid plays an antiproliferation and apoptosis role by regulating the expression of apoptosis-related proteins in gastric tumor cells, inducing cell-cycle arrest and autophagy, and has a better antitumor effect than 5-FU in xenograft models. In vitro experiments have confirmed that usnic acid has significant effects that induce morphological changes, inhibit cell proliferation in a time- and dosage-dependent manner, block cell cycle, promote apoptosis, and induce autophagy. Meanwhile, in vivo experiments have demonstrated that usnic acid is significantly more effective in inhibiting tumor growth without affecting body weight and in regulating the amount of Bax and Bcl2 in tumor tissues than 5-FU alone [81].

Experimental and clinical evidence has shown that tumor growth is dependent on angiogenesis. One of the most important hallmarks in cancer development is the induction of angiogenesis. Treatment with small-molecule drugs that inhibit angiogenesis has become an effective strategy for anticancer therapy. The effect and mechanism of usnic acid on H22 tumor growth in mice have been investigated and the results show that usnic acid can inhibit the growth and angiogenesis of H22 tumor in mice by inhibiting the secretion of vascular endothelial growth factor (VEGF) and bFGF [87]. Another study has shown that usnic acid can strongly inhibit angiogenesis in vivo in chicken chorioallantoic membrane and mouse corneal angiogenesis model induced by VEGF. In in vivo experiments, usnic acid not only significantly inhibits endothelial cell proliferation, migration, and tube formation but also induces morphological changes and apoptosis in endothelial cells. Western blot analysis has shown that usnic acid can inhibit breast tumor angiogenesis and growth by inhibiting VEGFR2-mediated AKT and ERK1/2 signaling pathways [88].

Owing to the chiral configuration of usnic acid, some studies have focused on (−)-usnic acid. Several studies have found that (−)-usnic acid has inhibitory effects on Lewis lung cancer cells [89] and mouse sarcoma 180 cells [90], moderate inhibitory effects on the proliferation of human colorectal cancer HT-29 cells [91], and significant inhibitory effect on murine leukemia P388 and murine lymphocytic leukaemia Ll210 cells [92,93,94].

**Table 3:** Antitumor activities and mechanism of usnic acid.

Cell Lines	Mechanism	IC_50_	Year	Reference
Human gastric carcinoma cell lines BGC823	Suppress the proliferation of human gastric carcinoma cells by inducing cycle phase arrest, cell apoptosis, and autophagy.	236.55 µM	2018	[81]
Human gastric carcinoma cell lines SGC7901		618.82 µM	2018	[81]
Human lung carcinoma A549 cells	Inhibit cell growth involving G0/G1 phase cell-cycle arrest and induce cell death via mitochondrial membrane depolarization and induction of apoptosis in human lung carcinoma cells.	NA	2013	[20]
Human breast cancer cell line MCF7	Selective cytotoxic effects on HDBC and HDPC cells without damaging normal cells and inducing apoptotic cell death and G0/G1 cell-cycle arrest.	71.4 µM	2018	[82]
Human prostate cancer cell line LNCaP		77.5 µM	2018	[82]
Human colon carcinoma wild-type p53 HCT-116 p53+/+ cells	Effective anti-cancer against a wide range of various cell lines originating from different tissues. It can accumulate cells in S-phase at the expense of the G1/G0-phase. Promote apoptosis.	157.2 µM	2011	[83]
Human colon carcinoma wild-type p53 HCT-116 p53−/− cells		143.1 µM	2011	[83]
Human leukemia cell line U937	The proliferation can be inhibited in a dose-dependent and time-dependent feature. The apoptosis of U937 cell induced by usnic acid is related to Caspase-dependent mitochondrial pathway.	90.90 μmol/L (24 h), 54.08 μmol/L (48 h)	2020	[84]
Human osteosarcoma cell line MG-63		103.00 μmol/L (24 h), 90.48 μmol/L (48 h)	2020	[84]
Human melanoma cell line A375		139.48 μmol/L (24 h), 65.39 μmol/L (48 h)	2020	[84]
Human prostate cancer cells PC-3M	Inhibition of DNA replication and RNA transcription of tumor cells, interfering with DNA synthesis, which eventually lead to the slowdown of proliferation rate of prostate cancer cells or accelerating the apoptosis of tumor cells.	NA	2005	[85]
Human lung carcinoma A549 cells	Inhibit PD-L1 protein synthesis by reducing STAT3 and RAS pathways cooperatively, induce MiT/TFE nuclear translocation through the suppression of mTOR signaling pathways, and promote the biogenesis of lysosomes and the translocation of PD-L1 to the lysosomes for proteolysis; Inhibit cell proliferation, angiogenesis, migration, and invasion, respectively, by downregulating PD-L1, thereby inhibiting tumor growth.	NA	2021	[86]
Human cervical cancer HeLa cells		NA	2021	[86]
Human cervical cancer SiHa cells		NA	2021	[86]
Human cervical cancer CaSKi cells		NA	2021	[86]
Mouse hepatocellular carcinoma cell line H22	Inhibitory effect on usnic acid on VEGF and bFGF.	NA	2016	[87]
Human umbilical vascular endothelial cells	Suppress Bcap-37 breast tumor growth and angiogenesis without affecting mice body weight in mouse xenograft tumor model; Inhibit endothelial cell proliferation, migration and tube formation. Induce morphological changes and apoptosis in endothelial cells in vitro; Block vascular endothelial growth factor receptor (VEGFR) 2 mediated extracellular signal-regulated protein kinases 1 and 2 (ERK1/2) and AKT/P70S6K signaling pathways in endothelial cells.	NA	2012	[88]
Human breast tumor cell line Bcap-37		NA	2012	[88]
Lewis lung carcinoma cells	/	NA	1975	[89]
S180 Bearing Mice	/	Inhibition rate: 75.1% (50 mg/kg) 66.6% (80 mg/kg) 69.1% (120 mg/kg)	1996	[90]
Human colorectal cancer HT-29 cells	/	55 µM	2013	[91]
Murine lymphocytic leukaemia L1210 (ATCC CCL 219)	The cytotoxic activity of usnic acid against cancer cell lines can be improved by its conjugation to a polyamine chain. The amine conjugation may not alter fundamentally the mode of action of usnic acid since both the parent compound and its derivative appeared to be apoptosis-inducing agents.	26.4 µM	2008	[92] *
Murine lymphocytic leukaemia L1210 (ATCC CCL 219)	Usnic acid was the only compound to display a moderate cytotoxic activity on various cancer cell lines. It was shown to induce apoptosis of murine leukaemia L1210 cells in a dose-and time-dependent manner.	6 μg/mL	2004	[93]
Lewis lung carcinoma	/	NA	1979	[94]
Murine leukemia P388 cells		NA	1979	[94]

*: Usnic-acid–amine conjugates; NA: Not Available.

Changes in people’s living environment, increases in living pressure, and the influence of economic and environmental factors have contributed to the increase in the incidence of cancer. Pharmacological intervention is an essential link in the treatment of cancer and the use of reasonable and effective anticancer drugs can help patients achieve long survival times. Usnic acid has been found to inhibit the proliferation of various cancer cells and has good research prospects.

### 2.4. Antioxidant and Photoprotection Effects

Oxidative stress refers to a state of imbalance between oxidation and antioxidant effects in the body and is mostly attributed to oxidation, a negative effect produced by free radicals in the body and considered an important factor in aging and disease.

Fernández-Moriano et al. [95] studied the mechanism of usnic acid and the cytoprotective properties in lichens of *Usnea* in vitro on the basis of antioxidant activity; they found that usnic acid can provide significant protection against H_2_O_2_-induced cytotoxic damage and reverse changes in apoptosis and in the markers of oxidative stress, including caspase-3 activity, ROS production, glutathione system activity, and lipid peroxidation level. This effect is mediated by a significant enhancement in the expression of intracellular phase II antioxidant enzymes and the Nrf2 cytoprotective pathway might be involved in this process.

Erfani et al. [96] investigated the neuroprotective effects of usnic acid on apoptotic cell death, neuroinflammation, antioxidant enzyme activity, and oxidative stress level after transient cerebral ischemia reperfusion; they provided significant evidence that usnic acid can treat cerebral ischemia injury in animal models as a neuroprotective agent. Their results showed that usnic acid can significantly alleviate memory impairment after transient global cerebral ischemia. Moreover, usnic acid can significantly reduce CA1 cell death by reducing caspase-3 activity, attenuate the neuroinflammation-mediated activation of microglia and astrocytes, and improve the antioxidant defense system in the hippocampus and spatial memory impairment after ischemia-induced neuronal injury. Usnic acid can also significantly inhibit lipid peroxidation and improve the antioxidant system by increasing the levels of SOD and glutathione (GSH) enzymes after cerebral ischemia in animal models.

In Turkey, *Usnea,* as folk medicine, is used to treat gastric ulcers. The antioxidant effects of usnic acid were studied in rats with indomethacin-induced gastric ulcers [32,97,98]; all doses of usnic acid were found to be more gastric protective than the H_2_ receptor blocker ranitidine. Superoxides generated by peroxidase in tissues may damage cell membranes and gastric tissues by increasing lipid peroxidation. Usnic acid can significantly increase the levels of SOD, GSH, and GPX and decrease the levels of LPO. Meanwhile, usnic acid can increase the activity of cNOS and cut down the activity of iNOS. All these results indicated that the gastroprotective effect of usnic acid can be attributed to its reducing effects on oxidative damage and its inhibitory effect on neutrophil infiltration of gastric rat tissues.

In addition, it was found that usnic acid can simultaneously promote oxidation and oxidation resistance under UVB light irradiation [28]. Cells exhibited high survival rates and normal metabolism at 1 × 10^−8^ mol/L and 1 × 10^−6^ mol/L usnic acid and 0.1 J/cm^2^ of UVB light dose, compared with a control group. At higher UVB dose (up to 14 J/cm^2^) and usnic acid concentration (1 × 10^−4^ mol/L), the survival rates of the cells were lower. The pro-oxidation activity of usnic acid is weak at low concentrations and physiological UVB doses. At 0.1 J/cm^2^, usnic acid exhibits a significant antioxidant function. This phenomenon suggests the potential application of usnic acid to clinical practice and cosmetics.

Natural substances extracted from lichens have been tested in vivo and in vitro as possible UV filters and the protective factors of natural substances have been compared with those of Nivea sunscreen spray LSF 5, octyl methoxycinnamate, and 4-tert-butyl-4′-dibenzoylmethane [29]; the results showed that usnic acid was the best UVB filter and its body-protective factors were similar to those of Nivea sunscreen spray LSF 5. In another study, after 21 days of exposure to natural sunlight, four of five photodegraded derivatives of usnic acid showed significant protective activity against UV and lower toxicity to human liver L02 cells and melanocytes than usnic acid [27].

In the course of evolution, many plants have gradually derived components with photoprotective ability and these components can effectively resist UV irradiation. Usnic acid and some of its derivatives have significant antioxidant functions, with potential applications in related industries.

### 2.5. Wound Healing

Wound healing is a complex process divided into three phases: inflammation, proliferation, and remodeling. These involve a variety of coordinated cellular activities, such as migration to wound areas, proliferation, and extracellular matrix deposition and remodeling.

Different concentrations of sodium usnic acid were added to L929 fibroblasts in vitro for the study of its effect on fibroblast proliferation [99]; the results showed that sodium usnic acid can promote wound healing, but not by stimulating the proliferation of fibroblasts. Sodium usnic acid can decrease inflammatory cells and promote the proliferation of fibroblasts and granulation tissues and vascular regeneration in wounds [25]. Moreover, it can promote the early generation of epithelial cells, formation of a well-organized collagen band, and epidermal keratinization and significantly increase VEGF level.

Pagano et al. [100] investigated different adhesive polymer films that improve the bioavailability of usnic acid in injury body; their study was based on the following: excellent inhibition ability of usnic acid against the common pathogens of burn wounds, such as Gram-positive bacteria and anaerobes [16,58,63,64,65,66,67], the anti-inflammatory effect of usnic acid [60], and the ability of usnic acid to stimulate keratinocyte monolayers to promote wound closure.

The effects of the five main polyketones in lichens, including (+)-usnic acid, on cell proliferation or wound healing have been investigated [24]. In this study, neutral red uptake and crystal violet cytotoxicity assays using MM98 malignant mesothelioma cells, A431 vulvar carcinoma cells, and HaCaT keratinocytes showed that usnic acid is highly toxic. However, usnic acid significantly stimulated wound closure in keratinocyte monolayers at subtoxic doses. This result suggests that usnic acid can be used to prevent hyperproliferative syndrome and promote tissue regeneration. Hypertrophic scar is a common fibroblast proliferation disease, characterized by the overexpression of collagen and excessive deposition of extracellular matrix in healing wounds caused by deep burns, inflammatory reactions, and trauma [101]. Inhibiting angiogenesis is an effective strategy for anti-hypertrophic scar treatment [102,103,104]. Moreover, usnic acid can significantly inhibit hypertrophic scars and scar angiogenesis and considerably reduce the height, color, and scar elevation index of a scar and collagen tissue accumulation markedly improves [105,106]. In vitro experiments have shown that usnic acid can inhibit the proliferation, migration, and formation of human umbilical vascular endothelial cells and inhibit the proliferation of scar fibroblasts. Bruno et al. [23] synthesized several enamine derivatives obtained from usnic acid; some derivatives with low cytotoxicity and high wound healing properties were used to promote wound healing or facilitate anti-aging skin preparation.

Owing to the increasing requirements of injured skin repair after trauma or surgery, many existing methods can no longer meet the needs of people and novel repair methods are constantly emerging in the market. Usnic acid can promote tissue regeneration and prevent excessive proliferation of wounds and can exert its effects at multiple stages of wound healing. How to make usnic acid safe and effective in promoting wound healing deserves further study.

### 2.6. Others

Verotta et al. [107] discovered that usnic acid is a weak antimalarial drug and exerts its effects at micromolar concentrations. Bruno et al. [108] combined usnic acid with dihydroartemisinin and found that the antimalarial activity of usnic acid is similar to artesunate, indicating that usnic acid has some activity against blood parasites in vitro and in vivo.

## 3. Toxicity

Although usnic acid has a variety of active effects, it has many adverse reactions, such as liver dysfunction and allergy contacting dermatitis, thus, attracting great interest.

### 3.1. Hepatotoxicity

Drug-induced liver injury (DILI) is one of the most-common liver diseases in clinical practice. Its incidence is increasing annually and ranks second to that of viral hepatitis and fatty liver disease. According to World Health Organization statistics, DILI has become the fifth-leading cause of death worldwide [109]. In the preclinical and clinical research stages of traditional Chinese medicine and natural medicine, hepatotoxicity has become one of the main reasons for drug development failure and drug withdrawal from the market. DILI induced by hepatotoxicity has become a problem worthy of special attention in drug discovery, particularly of botanical drugs.

#### 3.1.1. Liver Injury and Mechanism

Nutritional supplements for weight loss seem to be the most-commonly used and best-selling products. Oxidative uncouplers are growing in popularity, since they have been touted as “Can magically maintain muscle mass while accelerating fat loss”. A study has shown that usnic acid can act as an uncoupler of oxidative phosphorylation in mouse liver mitochondria [110]. This function may be one of the reasons that usnic acid is used in fat-burning supplements.

Similar conclusions have been drawn from the results of a study that examined the hepatotoxic effects of usnic acid on rats, isolated rat hepatocytes, and isolated rat liver mitochondria [111]; the study used glutamic acid with malic acid or succinic acid used as substrates to stimulate mitochondrial respiration and activate ATPase activity. The results indicated that low concentrations of usnic acid (0.15–6 μM) showed uncoupling activity in isolated rat liver mitochondria and high doses (1 mM) of usnic acid can induce the loss of cell membrane integrity in isolated rat hepatocytes by increasing the release of intracellular AST and ALT; meanwhile, lipid peroxidation and aniline hydroxylase activity increased and GSH content decreased in the cells. Reactive metabolites are speculated to be involved in the hepatotoxic effects of high doses of usnic acid.

However, several reports have shown that individuals suffer from severe hepatonecrosis, fulminant hepatic failure, and other toxicity effects after taking usnic acid or dietary supplements containing usnic acid [12,13,14,40,41,42,43]. Several reports have suggested that usnic acid in Lipokineti causes hepatotoxicity and usnic acid can cause moderate hepatic injury in normal rats in a dose-dependent manner [2,112,113]. Han et al. [114] determined the toxicity of usnic acid and evaluated whether usnic acid causes hepatotoxicity induced by Lipokineti; 5 mM usnic acid can cause 98% necrosis and no apoptosis was detected in primary mouse hepatocytes within 16 h. The mechanism might be similar to rotenone, which directly inhibits mitochondrial function, and lead to increases in active oxygen production by electron transport chain and, ultimately, cell death. This inhibitory effect of usnic acid on mitochondria corresponds to a decrease in ATP levels in hepatocytes. The direct inhibition and uncoupling of oxidative phosphorylation by usnic acid have been observed in isolated liver mitochondria and oxidative stress appears to be the critical factor of hepatotoxicity induced by usnic acid. An increase in the level of hydrogen peroxide produced by mitochondria when the respiratory chain is inhibited by usnic acid initiates oxidative stress and disrupts the normal metabolic processes of the cell.

The cytotoxicity of nine polyamine derivatives of usnic acid was evaluated in mouse and human cancer cell lines. Its cytotoxicity against cancer cells can be mitigated by combining usnic acid with the amino group [92]. The polyamine derivative of usnic acid showed significant cytotoxicity in L1210 cells, while their activity did not seem to depend on the polyamine transport system (PTS) and did not fundamentally change the apoptosis-inducing mode of usnic acid and its derivatives. The relevant conclusions require further investigation.

The roles of oxidative stress and Nrf2 signaling pathway in the usnic-acid-induced cytotoxicity of HepG2 cells have been investigated. Treatment with usnic acid for 24 h can result in DNA damage and S-phase cell-cycle arrest in a concentration-dependent manner [115]. However, short-term treatment, such as 6 h, can significantly increase Nrf2 protein level and promote Nrf2 translocation to the nucleus, upregulate the activity of antioxidant response element luciferase reporter, and induce the expression of Nrf2 regulatory targets, including glutathione reductase, glutathione S-transferase, and NAD(P)H quinone oxidoreductase-1. Furthermore, Nrf2 knockout by shRNA can aggravate DNA damage and cytotoxicity induced by usnic acid. These results suggest that usnic acid can induce DNA-damage-induced apoptosis and cell-cycle arrest and the Nrf2 signaling pathway is activated in usnic-acid-induced cytotoxicity.

The mechanism of hepatotoxicity of usnic acid in normal human L02 hepatocytes and ICR mice was investigated by using a variety of techniques, including immunoblot and siRNA transfection [116]; catalase expression decreased in a dose-dependent manner and the protein expressions of glutathione reductase, glutathione s-transferase, and glutathione peroxidase significantly increased in normal human L02 hepatocytes after usnic acid treatment. Usnic acid did not induce the activation of caspase-3, caspase-1 or gasdermin-D, and pyroptosis, the expression of porimin significantly increased, and cytotoxicity in porimin-silenced L02 cells. Nevertheless, usnic acid induced an infiltration of L02 cell death by increasing porin and forming irreversible membrane pores.

Most cellular studies have shown that usnic acid causes cell necrosis and affects mitochondrial function [34]. Some researchers believe that when cytochrome P450 1A (CYP1A) is inhibited, the decreased metabolism of usnic acid causes its accumulation and, thus, leads to the excessive inhibition of mitochondrial respiration, ATP deficiency, and cell necrosis [117]. In other words, some CYP enzyme inhibitors may increase the cytotoxicity of usnic acid to rat hepatocytes and usnic acid is more toxic than its metabolites and exerts a metabolic detoxification effect. However, some researchers believe that usnic acid decreases the GSH level in hepatocytes and inhibits ATP synthesis in mitochondria unrelated to apoptosis but induces oncosis [27]. Overall, the cause of hepatotoxicity caused by usnic acid has not been determined.

#### 3.1.2. Dose Dependence and Species Differences in Hepatotoxicity

After the oral administration of 500 and 1000 mg/kg usnic acid to male Wistar albino rats, no hepatotoxicity effects were observed after 24 h [97]; however, hepatotoxicity was observed after a high dose of usnic acid was administered (2000 mg/kg). These results suggest, to some extent, that the hepatotoxicity of usnic acid might be dose dependent and occur only at certain concentrations.

Usnic acid is toxic to some herbivorous insects, snails, and mammals, including elk, sheep, rabbit, rat, and human [118,119,120,121,122]. However, almost no animals can resist it. The Yunnan snub-nosed monkey (*Rhinopithecus bieti*) is a highly endangered species endemic to China and a primate with the highest distribution altitude [123]. Earlier studies suggested that the Yunnan snub-nosed monkey was a primate that fed mainly on *Usnea* [48]. However, significant differences in the food composition have been found among different Yunnan snub-nosed monkey populations in different habitats and are affected by altitude, climate, habitat type, and plant or animal species [48,52,124,125,126]. In the distribution areas of Yunnan snub-nosed monkey, *Usnea* is a food resource with wide distribution and high biomass and can be ingested throughout the year. The consumption of *Usnea* by Yunnan snub-nosed monkey has been observed throughout the year in various observational studies [48,52,124]. In the recipes of Yunnan golden snub-nosed monkeys from various populations, the proportion of *Usnea* consumption is approximately 50.6%–82.1% annually [48,124,126,127]. A study based on differences in the gut microbial community structure of Yunnan snub-nosed monkeys at different seasons showed that the average relative abundance of *Bacteroidetes*, *Proteobacteria*, and *Firmicutes* changed with season. This phenomenon suggests that the species of the gut microbiota in Yunnan snub-nosed monkeys played an important role in adapting to seasonal changes in food resources [128].

Reindeer is another animal that can consume lichens as its primary food, which are rich in usnic acid as their main food in the food-lacking winter [129,130]. Preliminary studies showed that usnic acid can be decomposed or degraded by microorganisms in the rumen of reindeer with the potential to be metabolized [55]. In free-ranging reindeer fed with lichens as their natural winter diet, the bacterial populations in the rumen were dominated by *Bacteroides*, *Fibrobacter*, *Streptococcus*, and *Clostridium*, and *Streptococcus* and *Clostridium* were predominant in reindeer fed with a pure lichen diet [130]. These microbial species in the rumen of reindeer have some similarities to those in Yunnan snub-nosed monkey; these similarities may be the reasons why both of them can feed on lichens rich in usnic acid without being affected by its toxicity.

The toxicity of any exotic matter is closely related to its dose. Therefore, when studying the toxicity of drugs, the dose should be paid attention to and the toxicity caused by exceeding the tolerable dose should arouse vigilance and attention. However, the specific mechanisms underlying the species differences in hepatotoxicity induced by usnic acid are a very interesting topic, which is worthy of further investigation and study.

### 3.2. Contact Dermatitis

Data on allergic dermatitis caused by human exposure to usnic acid are limited, although the association between lichens and contact dermatitis has been suspected as early as the beginning of the last century [131]. Usnic acid may be an occupational allergen in forestry workers and rural outdoor workers [132,133] and it may cause photosensitive contact dermatitis. Usnic acid may have a cross-reaction with oak moss, which is considered the main allergen in mixtures of spices and is a special contact allergen [134]. Allergic contact eczema can occasionally be caused by usnic acid in natural deodorants or deodorizing sprays [135,136]. Vaginal ovules, an antimicrobial agent containing usnic acid, may cause allergic dermatitis. However, in the year before this case was reported, none of the 24 patients who had undergone an usnic acid skin test showed allergic reactions [39]. A significant allergic reaction associated with usnic acid in guinea pigs was reported in 1993 [137]. A case report of contact dermatitis due to usnic acid in dentists was reported; it was the first report of occupational contact dermatitis caused by usnic acid in office workers and the researcher believed that usnic acid is a potential airborne or photosensitive allergen [138].

## 4. Metabolism and Pharmacokinetics of Usnic Acid In Vivo and In Vitro

Drug metabolism refers to the process in which the chemical structure of a drug changes under the actions of multiple drug-metabolizing enzymes (especially liver drug enzymes) in the body.

The protein binding of usnic acid to rabbit plasma and purified bovine serum albumin in vitro were studied. Usnic acid can highly bind to proteins, with a 99.2% binding rate, and the bonds were stable at an usnic acid concentration of 1–5 μg/mL [139]. After an intraperitoneal injection of 25 mg/kg usnic acid in rats, the concentration of usnic acid in the liver, lungs, and whole blood was slightly higher than that in the plasma. Another report found that the binding rate of usnic acid was 99.76% in human plasma and 99.49% in rat plasma [140], similar to previous studies [139].

Ultra-high-performance liquid chromatography–triple/time-of-flight mass spectrometry was used in analyzing and determining the metabolites of usnic acid in rats, so as to reveal the metabolic characteristics of usnic acid [141]. A total of 36 metabolites, including 27 phase I metabolites and 9 phase II metabolites, was tentatively detected after the oral administration of usnic acid in rats; 33 metabolites were detected in the urine, 8 in plasma, and 16 in bile. The metabolic pathways of usnic acid may include oxidation, reduction, dihydroxylation, glycine conjugation, glucuronidation, *N*-acetylcysteine conjugation, and methylation, oxidation reaction and glucuronic acid conjugation are the main metabolism types in phases I and II, respectively. Usnic acid can be metabolized rapidly and thoroughly in the body and excreted mainly as metabolites. The results have enhanced understanding of the bio-transformations and pharmaceutical applications of usnic acid.

Reactive metabolites play an important role in acetaminophen-induced liver injury [142]. Subsequent studies have shown that most drugs result in the relatively high incidence of liver injury produced by reactive metabolites [143]. Reactive metabolites are also involved in most idiosyncratic drug reactions and some drugs that are toxic to the liver may act by producing reactive metabolites that damage the liver. The potential role of the reactive metabolites of usnic acid in hepatotoxicity has been suggested previously [111]. Trapping assay with glutathione was used in identifying possible novel metabolites of usnic acid to speculate the formation of reactive metabolites of usnic acid in human, rat, and mouse liver microsomes to determine the biotransformation process of usnic acid enantiomers to reactive products [144]. Approximately 50 μM (+) or (−)-usnic acid was incubated with a mixture of human, rat, and mouse liver microsomes and glutathione (the trapping nucleophile). The results showed that both enantiomers of usnic acid produced two reactive metabolites through hydroxylation and dehydrogenation, which were the enantiomers of each other. The fragmentation patterns of these products were consistent with the characteristics of glutathione adducts. This may partially explain the toxicity of usnic acid; that is, the formation of reactive metabolites of usnic acid may be one of the mechanisms of usnic-acid-induced liver injury. In addition, the semiquantitative analysis of the metabolites showed that the peak areas of the metabolites produced by the two enantiomers of usnic acid in different microsomes varied. The different proportions of usnic acid metabolites produced in human, rat, and mouse liver microsomes imply that rodent models are less useful in the study of hepatoxicity dependent on usnic acid metabolite, and the extrapolation of experimental results from rodents to humans should be performed with caution.

The cytochrome P450 oxidative metabolism enzyme system is one of the key enzymes involved in drug metabolism. Three hydroxylation products and two glucuronidation products of usnic acid in in vitro experiments were discovered; the oxidative metabolism reaction was mainly carried out by CYP1A2 and the glucuronidation reaction was carried out by UGT1A1 and UGT1A3 [140]. This study demonstrated that usnic acid is a potent inhibitor of CYP2C19 and CYP2C9, a weak potent inhibitor of CYP2C8 and CYP2C18, and a relatively weak inhibitor of CYP2D6. Another study found that the metabolism of usnic acid decreased and resulted in self-accumulation when CYP1A was inhibited and, ultimately, in cell death [117]. This phenomenon suggests that usnic acid is more toxic than its metabolites to rat hepatocytes when some CYP enzymes are inhibited and usnic acid exerts metabolic detoxification effects.

The pharmacokinetic changes were observed 48 h after the administration of 5 and 20 mg/kg body weight of usnic acid intravenously and orally in normal male rabbits [145,146]. The pharmacokinetic profile of usnic acid was described as a three-compartment model. After the intravenous and oral administration of 5 and 20 mg/kg usnic acid, the average terminal half-life in the plasma was about 10.7 ± 4.6 and 18.9 ± 2.9 h, respectively. The pharmacokinetic parameters obtained from compartmental and noncompartmental models were close and the bioavailability was about 77.8%. The pharmacokinetic behavior of usnic acid and phenolic acids, such as barbatic acid, diffractaic acid, and evernic acid, was studied in vivo in another study after intravenous and oral administration of pure usnic acid and extract from *U. longissima*; the results showed that the absolute bioavailability of usnic acid was 69.2% and 146.9% after oral administration of usnic acid and ethanolic extract, respectively [147], indicating that the other coexisting ingredients in the extract can promote the absorption of usnic acid or inhibit its metabolism and elimination, thus, affecting its pharmacokinetic behavior in vivo.

To improve liposolubility, absorption, and bioavailability and reduce adverse effects of usnic acid, different dosage forms, such as nanosuspensions, have been used. Compared with usnic acid i.g. rats, nanosuspension administration rats showed shortened maximum plasma time (T_max_) and prolonged terminal elimination half-life (t_1/2_). The maximum plasma concentration (C_max_) and plasma concentration versus the time curve from zero to time t (AUC_0–t_) increased and oral bioavailability increased 3.09-fold [148]. In another study, usnic acid was prepared as a phospholipid complex. The relative bioavailability of usnic acid increased from 109.67% to 177.83% through the phospholipid complex [149]. Meanwhile, at the measured administration concentrations, the tissue distribution of usnic acid in rats was also changed due to changes in dosage form. The transport of drugs to tissues is mainly determined by the concentration of free drugs in the blood and the affinity of drugs and tissues. However, the variation patterns of drug concentrations among tissues were less consistent at doses of 11.7, 17.5, and 35.0 mg/kg. This finding indicates that the distribution of usnic acid in various tissues may be related to the administered dose and may be caused by individual differences in rats.

Metabolic and toxicity studies have shown that usnic acid can strongly bind to proteins and has high bioavailability, which may be the reasons for the hepatotoxicity of usnic acid in many mammals. Drugs usually have detoxification and toxification biotransformation pathways. The results of a single study in an in vitro model have not fully demonstrated the toxicity of the observed reactive metabolites. Yunnan snub-nosed monkey and reindeer are the only two mammals known to decompose or metabolize usnic acid in vivo, but neither of their in vivo processes is clear. The in vivo process may be related to gastrointestinal microorganisms. Currently, many studies on the pharmacological activity of usnic acid have focused on how to reduce its toxicity on the basis of rational development and utilization of usnic acid activity. Studies on the metabolism and in vivo process of usnic acid are still insufficient and deserve further study to provide data for its safe use. The proposal of a novel mechanism of usnic-acid-induced hepatotoxicity can provide some new enlightenment for the development of safe usnic acid derivatives.

## 5. Conclusions

We systematically summarized studies on the biological activities, toxicity, metabolism, and pharmacokinetics of usnic acid in this review. *Usnea* species have been used as antimicrobials since 101 B.C. in traditional Chinese medicine and been recorded as treatments for liver detoxification, malaria, snake bite, wound infection, tuberculosis, and cough. Before penicillin was discovered, usnic-acid-rich lichen extracts had already been widely used in many products worldwide. According to current research, usnic acid, as the main active chemical constituent in *Usnea,* exhibits various biological activities, including anti-inflammatory, antibacterial, antiviral, antitumor, antioxidant, and photoprotection, as well as wound-healing properties.

Studies on usnic acid have mainly focused on its biological activities, whereas studies on the mechanism of hepatotoxicity and metabolism remain limited. Some studies conducted structural modifications or made attempts to discover novel drug-loaded forms of usnic acid and reduce its hepatotoxicity on the basis of retaining its biological activity. Meanwhile, the use of usnic acid in food, medicine, and other industries has been affected and limited due to its hepatotoxicity.

Drugs often work in an organism through multiple pathways rather than a single pathway. Although usnic acid is one of the earliest commercially used lichen compounds and has been used for many years, research on usnic acid still suffers from many limitations. How to prevent hepatotoxicity is a noteworthy issue. Studies on metabolism and the influencing factors of usnic acid in different animals have become another issue worthy of further study. Further studies are needed to determine its efficacy and safety. The toxicological mechanism needs to be improved for the safe, reasonable, and effective use of usnic acid in food and clinical practice.

## Figures and Tables

**Table 1:** Anti-inflammatory activities and mechanism of usnic acid.

Model	Mechanism or Effect	Concentration	Year	References
Acute and chronic inflammation rat models	Reduce the swelling of rat foot induced by carrageenan in dose dependent. Only effect at 100 mg/kg.	25 mg/kg, 50 mg/kg, 100 mg/kg, p.o.	2000	[18]
Lipopolysaccharide (LPS) activated RAW264.7 macrophages	Inhibit the express of TNF-α and iNOS, possibly through suppression of nuclear translocation of NF-κB p65 and I-κBα degradation; Inhibit LPS-induced TNF-α accumulation and NO production in a dose-dependent manner.	0.5–400 μM; IC_50_: 4.7 μM (TNF-α), 12.8 μM (NO)	2008	[59]
Lipopolysaccharide (LPS) activated RAW264.7 macrophages	Down-regulating iNOS, COX-2, IL-1β, IL-6 and TNF-α, COX-2 gene expression through the suppression of NF-κB activation and increasing anti-inflammatory cytokine IL-10 and anti-inflammatory mediator HO-1 production.	10 μg/mL, 50 μg/mL and 100 μg/mL	2011	[60]
1-methyl-4-phenyl-1,2,3,6-tetrahydropyridine (MPTP) induced Parkinson’s disease model in Male C57BL/6 mice	Inhibit MPP^+^-induced glial activation in primary astrocytes by blocking NF-κB activation.	5 or 25 mg/kg dissolved in phosphate buffered saline (PBS) containing 5% Tween-80 (i.p.)	2020	[61]
Tau protein derived hexapeptide AcPHF6 model	Inhibit the aggregation of full-length 2N4R tau protein by a heparin-induced mechanism.	10 μM	2020	[62] *
Human neuroblastoma SK-N-SH cell line (SH-SY5Y)	Exert no significant hepatotoxicity, showed low hepatotoxicity at 40 μM.	10–40 μM	2020	[62] *
Human hepatocyte cells (LO2) cells	Exert no significant hepatotoxicity, showed low hepatotoxicity at 40 μM.	10–40 μM	2020	[62] *
Murine microglial BV2 cells	Reduce NO release in Lipopolysaccharide-stimulated mouse microglia BV2 cells.	10–40 μM	2020	[62] *
Adult male SD rats	Enhance the cognitive ability of okadiac acid induced AD model rats.	5 mg/kg, 10 mg/kg	2020	[62] *

*: Usnic acid derivatives, synthesized from the reaction of usnic acid with the corresponding hydrazines and hydrazides.

**Table 2:** Antibacterial and antiviral activities of usnic acid.

Bacterial Strain	MIC or Others	Year	Reference
*Mycobacterium tuberculosis* H37Rv ATCC 27294	12.25 µg/mL	2010	[16]
*Isoniazid resistant Mycobacterium tuberculosis*	1.56 µg/mL	2010	[16]
*Rifampicin resistant Mycobacterium tuberculosis*	12.5 µg/mL	2010	[16]
*Streptomycin resistant Mycobacterium tuberculosis*	6.25 µg/mL	2010	[16]
*Mycobacterium fortuitum* ATCC 35931	50 µg/mL	2010	[16]
*Mycobacterium chelonae* ATCC 946	25 µg/mL	2010	[16]
*Mycobacterium kansasii* ATCC 12478	12.5 µg/mL	2010	[16]
*Mycobacterium avium*	100 µg/mL	2010	[16]
*Staphylococcus aureus* ATCC 25923	6.2 µg/mL	2009	[58]
*Pneumococcus*	12.5 µg/mL	2009	[58]
*Pseudomonas aeruginosa*	/	2009	[58]
*Bacillus coli* ATCC 35218	/	2009	[58]
*Bacillus subtilis*	8 μg/mL	2011	[63]
*Bacillus cereus*	8 μg/mL	2011	[63]
*Staphylococcus aureus*	31 μg/mL	2011	[63]
*Escherichia coli*	31 μg/mL	2011	[63]
*Propionibacterium acnes* FR 024/12-10	1 μg/mL	2007	[65]
*Propionibacterium acnes*	2 μg/mL	1995	[66]
Methicillin-susceptible *Staphylococcus aureus*	2–>16 μg/mL	1995	[66]
Methicillin-resistant, mupirocin-susceptible *Staphylococcus aureus*	4–16 μg/mL	1995	[66]
Methicillin-resistant, mupirocin-resistant *Staphylococcus aureus*	4–16 μg/mL	1995	[66]
*Mycobacterium aurum*	32 μg/mL	1998	[67]
*Bacillus subtilis* 78A	NA	2018	[68] *
*Pseudomonas fluorescens* BKM CR-330	NA	2018	[68] *
*Aspergillus flavus*	NA	2000	[69]
*Aspergillus niger*	NA	2000	[69]
*Blue mould*	NA	2000	[69]
*Rhizopus*	NA	2000	[69]
*Bacillus subtilis*	NA	2000	[69]
*Bacillus coli*	NA	2000	[69]
*Staphylococcus albus*	NA	2000	[69]
*Lactobacilli*	NA	2000	[69]
*Baker’s yeast*	NA	2000	[69]
*Rhodotorula*	NA	2000	[69]
*Staphylococcus aureus* MTCC-96	25 μg/mL	2012	[70]
Methicillin-resistant *Staphylococcus aureus*	25–50 μg/mL	2012	[70]
*Escherichia coli*	20 μg/mL	2014	[71]
*Vibrio harveyi*	20 μg/mL	2014	[71]
*Bacillus subtilis*	0.5 μg/mL	2014	[71]
*Staphylococcus aureus*	1.0 μg/mL	2014	[71]
*Mycobacterium abscessus* ATCC 19977	18.15 µM	2018	[72]
*Mycobacterium abscessus* subsp. *Abscessus* AT 07	9.07 µM	2018	[72]
*Mycobacterium abscessus* subsp. *Abscessus* AT 46	9.07 µM	2018	[72]
*Mycobacterium abscessus* subsp. *bolletii* AT 52	9.07 µM	2018	[72]
Methicillin-resistant *Staphylococcus aureus* ATCC 43300, AQ 004, AQ 006, AQ 007, AQ 012	1–8 μg/mL	2012	[73]
Herpes simplex type 1 virus	7.5 μg per disc leads to over 4 mm inhibite zone	1999	[76]
Polio type 1 virus	30 μg per disc leads to over 4 mm inhibite zone	1999	[76]
Human immunodeficiency virus RF	/	NA	[77]
H1N1 influenza virus pdm09	ED_50_: 51.7 μM	2012	[22]
SARS-CoV-2 original strain	IC_50_: 7.99 μM	2022	[79]
SARS-CoV-2 alpha variant (UK, B.1.1.7)	IC_50_: 6.05 μM	2022	[79]
SARS-CoV-2 beta variant (South Africa, B.1.351)	IC_50_: 2.92 μM	2022	[79]
SARS-CoV-2 delta variant (India, B.1.617.2)	IC_50_: 7.17 μM	2022	[79]

*: *U. barbata* dry extracts (in terms of usnic acid) 0.24–0.6 mg/mL; NA: Not Available.

## Data Availability

Not applicable.

## References

[1] Choudhary M.I., Azizuddin, Jalil S., Atta-Ur-Rahman (2005). Bioactive phenolic compounds from a medicinal lichen, Usnea longissima. Phytochemistry.

[2] Guo L., Shi Q., Fang J.L., Mei N., Ali A.A., Lewis S.M., Leakey J.E., Frankos V.H. (2008). Review of usnic acid and Usnea barbata toxicity. J. Environ. Sci. Health, Part C Environ. Carcinog. Ecotoxicol. Rev..

[3] Zang M., Li X. (2011). Dictionary of the Families and Genera of Chinese Cryptogamic (Spore) Plants.

[4] Laxineimujila, Bao H., Tu L. (2013). Advance in studies on chemical constituents and pharmacological activity of lichens in Usnea genus. China J. Chin. Mater. Med..

[5] Huang Z., Huang L., Zhang Y., Lin Y. (2009). The Illustration of Common Medicinal Plants in Taiwan Vol. I.

[6] Prateeksha, Paliya B.S., Bajpai R., Jadaun V., Kumar J., Kumar S., Upreti D.K., Singh B.R., Nayaka S., Joshi Y. (2016). The genus Usnea: A potent phytomedicine with multifarious ethnobotany, phytochemistry and pharmacology. RSC Adv..

[7] Shukla V., Rawat G. (2010). Lichens as a potential natural source of bioactive compounds: A review. Phytochem. Rev..

[8] Srivastava P., Upreti D.K., Dhole T.N., Srivastava A.K., Nayak M.T. (2013). Antimicrobial property of extracts of indian lichen against human pathogenic bacteria. Interdiscip. Perspect. Infect. Dis..

[9] Jiangsu New Medical College (1977). Chinese Materia Medica Dictionary (The First Volume).

[10] Chinese Pharmacopoeia Committee (1999). Drug Standards of the Ministry of Public Health of the People’s Republic of China (Uygur Pharmaceutical Section).

[11] Chinese Herbalism Editorial Board (2004). State administration of traditional chinese medicine of the people’s republic of china. Chinese Materia Medica • Mongolian Medicine.

[12] Brown A.C. (2017). Liver toxicity related to herbs and dietary supplements: Online table of case reports. Part 2 of 5 series. Food Chem. Toxicol..

[13] Avigan M.I., Mozersky R.P., Seeff L.B. (2016). Scientific and regulatory perspectives in herbal and dietary supplement associated hepatotoxicity in the United States. Int. J. Mol. Sci..

[14] Felix S., Sara D., Eleonora P., Katja B., Beat A., Stephen L.L. (2009). Severe hepatotoxicity following ingestion of Herbalife^®^ nutritional supplements contaminated with Bacillus subtilis. J. Hepatol..

[15] Honda N.K., Pavan F.R., Coelho R.G., de Andrade L.S., Micheletti A.C., Lopes T.I., Misutsu M.Y., Beatriz A., Brum R.L., Leite C.Q. (2010). Antimycobacterial activity of lichen substances. Phytomedicine.

[16] Ramos D.F., Almeida D.S.P. (2010). Antimycobacterial activity of usnic acid against resistant and susceptible strains of Mycobacterium tuberculosis and non-tuberculous mycobacteria. Pharm. Biol..

[17] Okuyama E., Umeyama K., Yamazaki M., Kinoshita Y., Yamamoto Y. (1995). Usnic acid and diffractaic acid as analgesic and antipyretic components of Usnea diffracta. Planta Med..

[18] Vijayakumar C.S., Viswanathan S., Reddy M.K., Parvathavarthini S., Kundu A.B., Sukumar E. (2000). Anti-inflammatory activity of (+)-usnic acid. Fitoterapia.

[19] Backorova M., Jendzelovsky R., Kello M., Backor M., Mikes J., Fedorocko P. (2012). Lichen secondary metabolites are responsible for induction of apoptosis in HT-29 and A2780 human cancer cell lines. Toxicol. Vitr..

[20] Singh N., Nambiar D., Kale R.K., Singh R.P. (2013). Usnic acid inhibits growth and induces cell cycle arrest and apoptosis in human lung carcinoma A549 cells. Nutr. Cancer.

[21] Shtro A.A., Zarubaev V.V., Luzina O.A., Sokolov D.N., Kiselev O.I., Salakhutdinov N.F. (2014). Novel derivatives of usnic acid effectively inhibiting reproduction of influenza a virus. Bioorg. Med. Chem..

[22] Sokolov D.N., Zarubaev V.V., Shtro A.A., Polovinka M.P., Luzina O.A., Komarova N.I., Salakhutdinov N.F., Kiselev O.I. (2012). Anti-viral activity of (−)- and (+)-usnic acids and their derivatives against influenza virus A(H1N1)2009. Bioorg. Med. Chem. Lett..

[23] Bruno M., Trucchi B., Burlando B., Ranzato E., Martinotti S., Akkol E.K., Suntar I., Keles H., Verotta L. (2013). (+)-Usnic acid enamines with remarkable cicatrizing properties. Bioorg. Med. Chem..

[24] Burlando B., Ranzato E., Volante A., Appendino G., Pollastro F., Verotta L. (2009). Antiproliferative effects on tumour cells and promotion of keratinocyte wound healing by different lichen compounds. Planta Med..

[25] Zhang Z.H., Zheng Y., Huibai Y.L., Ma T., Song X., Zhao J., Gao L. (2018). The effects of sodium usnic acid by topical application on skin wound healing in rats. Biomed. Pharmacother..

[26] Lohezic-Le D.F., Legouin B., Couteau C., Boustie J., Coiffard L. (2013). Lichenic extracts and metabolites as UV filters. J. Photochem. Photobiol. B.

[27] Kwong S.P., Wang H.X., Shi L., Huang Z.L., Lu B., Cheng X.M., Chou G.X., Ji L.L., Wang C.H. (2020). Identification of photodegraded derivatives of usnic acid with improved toxicity profile and UVA/UVB protection in normal human L02 hepatocytes and epidermal melanocytes. J. Photochem. Photobiol. B.

[28] Kohlhardt-Floehr C., Boehm F., Troppens S., Lademann J., Truscott T.G. (2010). Prooxidant and antioxidant behaviour of usnic acid from lichens under UVB-light irradiation--studies on human cells. J. Photochem. Photobiol. B.

[29] Rancan F., Rosan S., Boehm K., Fernandez E., Hidalgo M.E., Quihot W., Rubio C., Boehm F., Piazena H., Oltmanns U. (2002). Protection against UVB irradiation by natural filters extracted from lichens. J. Photochem. Photobiol. B.

[30] Bayir Y., Odabasoglu F., Cakir A., Aslan A., Suleyman H., Halici M., Kazaz C. (2006). The inhibition of gastric mucosal lesion, oxidative stress and neutrophil-infiltration in rats by the lichen constituent diffractaic acid. Phytomedicine.

[31] Rabelo T.K., Zeidan-Chulia F., Vasques L.M. (2012). Redox characterization of usnic acid and its cytotoxic effect on human neuron-like cells (SH-SY5Y). Toxicol. Vitr..

[32] Halici M., Odabasoglu F., Suleyman H., Cakir A., Aslan A., Bayir Y. (2005). Effects of water extract of Usnea longissima on antioxidant enzyme activity and mucosal damage caused by indomethacin in rats. Phytomedicine.

[33] Salgado F., Albornoz L., Cortez C., Stashenko E., Urrea-Vallejo K., Nagles E., Galicia-Virviescas C., Cornejo A., Ardiles A., Simirgiotis M. (2017). Secondary metabolite profiling of species of the genus usnea by UHPLC-ESI-OT-MS-MS. Molecules.

[34] Araujo A.A., de Melo M.G., Rabelo T.K., Nunes P.S., Santos S.L., Serafini M.R., Santos M.R., Quintans-Junior L.J., Gelain D.P. (2015). Review of the biological properties and toxicity of usnic acid. Nat. Prod. Res..

[35] Ingolfsdottir K. (2002). Usnic acid. Phytochemistry.

[36] Romagni J.G., Meazza G., Nanayakkara N.P., Dayan F.E. (2000). The phytotoxic lichen metabolite, usnic acid, is a potent inhibitor of plant p-hydroxyphenylpyruvate dioxygenase. FEBS Lett..

[37] Sweetman S.C. (2009). Martindale: The Complete Drug Reference.

[38] Vartia K.O. (1973). The Lichens.

[39] Rafanelli S., Bacchilega R., Stanganelli I., Rafanelli A. (1995). Contact dermatitis from usnic acid in vaginal ovules. Contact Dermat..

[40] Yellapu R.K., Mittal V., Grewal P., Fiel M., Schiano T. (2011). Acute liver failure caused by ‘fat burners’ and dietary supplements: A case report and literature review. Can. J. Gastroenterol..

[41] Sanchez W., Maple J.T., Burgart L.J., Kamath P.S. (2006). Severe hepatotoxicity associated with use of a dietary supplement containing usnic acid. Mayo Clin. Proc..

[42] Durazo F.A., Lassman C., Han S.H., Saab S., Lee N.P., Kawano M., Saggi B., Gordon S., Farmer D.G., Yersiz H. (2004). Fulminant liver failure due to usnic acid for weight loss. Am. J. Gastroenterol..

[43] Favreau J.T., Ryu M.L., Braunstein G., Orshansky G., Park S.S., Coody G.L., Love L.A., Fong T.L. (2002). Severe hepatotoxicity associated with the dietary supplement LipoKinetix. Ann. Intern. Med..

[44] Frankos V. NTP Nomination for Usnic Acid and Usnea barbata Herb. https://ntp.niehs.nih.gov/ntp/htdocs/chem_background/exsumpdf/usnicacid_508.pdf.

[45] Stickel F., Shouval D. (2015). Hepatotoxicity of herbal and dietary supplements: An update. Arch. Toxicol..

[46] (2002). ‘Dietary supplement’ warning. FDA Consum..

[47] Caldwell J.P., Kim N.D. (1997). The response of the Intoxilyzer 5000 to five potential interfering substances. J. Forensic Sci..

[48] Kirkpatrick R. (1996). Ecology and Behavior of the Yunnan Snub-Nosed Langur (Rhinopithecus Bieti, Colobinae). Ph.D. Thesis.

[49] Mu W., Yang D. (1982). Preliminary observation on Rhinopithecus Bieti group, movement route and feeding habits of Yunnan snub-nosed monkey on the eastern slope of Baima Snow Mountain. Acta Theriol. Sin..

[50] Zhao W., Yang P., Shen Y., He X., He S., Si N., Su M., Shi F. (2009). Survey on the feeding habits and food resources of Yunnan snub-nosed monkey in Tacheng area in the south of Baima Snow Mountain Nature Reserve. Chin. J. Zool..

[51] Xiang Z.F., Huo S., Xiao W., Quan R.C., Grueter C.C. (2007). Diet and feeding behavior of Rhinopithecus bieti at Xiaochangdu, Tibet: Adaptations to a marginal environment. Am. J. Primatol..

[52] Wei D., Zhao Q. (2004). Rhinopithecus bieti at Tacheng, Yunnan: Diet and Daytime Activities. Int. J. Primatol..

[53] Li D.Y. (2010). Study on Activity Time Allocation, Nocturnal Behavior and Feeding Habits of Yunnan Snub-Nosed Monkey (Rhinopithecus Bieti) in Baima Snow Mountain Nature Reserve. Ph.D. Thesis.

[54] Sundset M.A., Kohn A., Mathiesen S.D., Praesteng K.E. (2008). Eubacterium rangiferina, a novel usnic acid-resistant bacterium from the reindeer rumen. Naturwissenschaften.

[55] Sundset M.A., Barboza P.S., Green T.K., Folkow L.P., Blix A.S., Mathiesen S.D. (2010). Microbial degradation of usnic acid in the reindeer rumen. Naturwissenschaften.

[56] Glad T., Barboza P., Mackie R.I., Wright A.D., Brusetti L., Mathiesen S.D., Sundset M.A. (2014). Dietary supplementation of usnic acid, an antimicrobial compound in lichens, does not affect rumen bacterial diversity or density in reindeer. Curr. Microbiol..

[57] Cook W.E., Raisbeck M.F., Cornish T.E., Williams E.S., Brown B., Hiatt G., Kreeger T.J. (2007). Paresis and death in elk (Cervus elaphus) due to lichen intoxication in Wyoming. J. Wildl. Dis..

[58] Zhao Y., Song D., Tao J., Zhang T. (2009). Preliminary study on antibacterial and anti-inflammatory effects of raw material and self-microemulsion of usnic acid. J. Emerg. Tradit. Chin. Med..

[59] Jin J.Q., Li C.Q., He L.C. (2008). Down-regulatory effect of usnic acid on nuclear factor-kappaB-dependent tumor necrosis factor-alpha and inducible nitric oxide synthase expression in lipopolysaccharide-stimulated macrophages RAW 264.7. Phytother. Res..

[60] Huang Z., Zheng G., Tao J., Ruan J. (2011). Anti-inflammatory effects and mechanisms of usnic acid. J. Wuhan Univ. Technol. Mater. Sci. Ed..

[61] Lee S., Lee Y., Ha S., Chung H.Y., Kim H., Hur J.S., Lee J. (2020). Anti-inflammatory effects of usnic acid in an MPTP-induced mouse model of Parkinson’s disease. Brain Res..

[62] Shi C.J., Peng W., Zhao J.H., Yang H.L., Qu L.L., Wang C., Kong L.Y., Wang X.B. (2020). Usnic acid derivatives as tau-aggregation and neuroinflammation inhibitors. Eur. J. Med. Chem..

[63] Sultana N., Afolayan A.J. (2011). A new depsidone and antibacterial activities of compounds from Usnea undulata Stirton. J. Asian Nat. Prod. Res..

[64] Lin Y. (2011). Research status of usnea. China Pharm..

[65] Weckesser S., Engel K., Simon-Haarhaus B., Wittmer A., Pelz K., Schempp C.M. (2007). Screening of plant extracts for antimicrobial activity against bacteria and yeasts with dermatological relevance. Phytomedicine.

[66] Lauterwein M., Oethinger M., Belsner K., Peters T., Marre R. (1995). In vitro activities of the lichen secondary metabolites vulpinic acid, (+)-usnic acid, and (−)-usnic acid against aerobic and anaerobic microorganisms. Antimicrob. Agents Chemother..

[67] Ingolfsdottir K., Chung G.A., Skulason V.G., Gissurarson S.R., Vilhelmsdottir M. (1998). Antimycobacterial activity of lichen metabolites in vitro. Eur. J. Pharm. Sci..

[68] Bazarnova Y., Politaeva N., Lyskova N. (2018). Research for the lichen Usnea barbata metabolites. Z. Naturforsch. C J. Biosci..

[69] Zhao X. (2000). Study on Bacteriostasis of usnic acid. Food Sci..

[70] Gupta V.K., Verma S., Gupta S., Singh A., Pal A., Srivastava S.K., Srivastava P.K., Singh S.C., Darokar M.P. (2012). Membrane-damaging potential of natural L-(−)-usnic acid in Staphylococcus aureus. Eur. J. Clin. Microbiol. Infect. Dis..

[71] Maciag-Dorszynska M., Wegrzyn G., Guzow-Krzeminska B. (2014). Antibacterial activity of lichen secondary metabolite usnic acid is primarily caused by inhibition of RNA and DNA synthesis. FEMS Microbiol. Lett..

[72] Ramis I.B., Vianna J.S., Reis A.J., von Groll A., Ramos D.F., Viveiros M., Da S.P. (2018). Antimicrobial and efflux inhibitor activity of usnic acid against mycobacterium abscessus. Planta Med..

[73] Segatore B., Bellio P., Setacci D., Brisdelli F., Piovano M., Garbarino J.A., Nicoletti M., Amicosante G., Perilli M., Celenza G. (2012). In vitro interaction of usnic acid in combination with antimicrobial agents against methicillin-resistant Staphylococcus aureus clinical isolates determined by FICI and DeltaE model methods. Phytomedicine.

[74] Scirpa P., Scambia G., Masciullo V., Battaglia F., Foti E., Lopez R., Villa P., Malecore M., Mancuso S. (1999). A zinc sulfate and usnic acid preparation used as post-surgical adjuvant therapy in genital lesions by Human Papillomavirus. Minerva Ginecol..

[75] Yamamoto Y., Miura Y., Kinoshita Y., Higuchi M., Yamada Y., Murakami A., Ohigashi H., Koshimizu K. (1995). Screening of tissue cultures and thalli of lichens and some of their active constituents for inhibition of tumor promoter-induced Epstein-Barr virus activation. Chem. Pharm. Bull..

[76] Perry N.B., Benn M.H., Brennan N.J., Burgess E.J., Ellis G., Galloway D.J., Lorimer S.D., Tangney R.S. (1999). Antimicrobial, antiviral and cytotoxic activity of New Zealand lichens. Lichenologist.

[77] Division of AIDS Anti-HIV/OI/TB Therapeutics Database. https://chemdb.niaid.nih.gov/CellularDetails.aspx?AIDSNO=028613&pn=1.

[78] Pinto R., Herold S., Cakarova L., Hoegner K., Lohmeyer J., Planz O., Pleschka S. (2011). Inhibition of influenza virus-induced NF-kappaB and Raf/MEK/ERK activation can reduce both virus titers and cytokine expression simultaneously in vitro and in vivo. Antivir. Res..

[79] Oh E., Wang W., Park K.H., Park C., Cho Y., Lee J., Kang E., Kang H. (2022). (+)-Usnic acid and its salts, inhibitors of SARS-CoV-2, identified by using in silico methods and in vitro assay. Sci. Rep..

[80] Ingelfinger R., Henke M., Roser L., Ulshofer T., Calchera A., Singh G., Parnham M.J., Geisslinger G., Furst R., Schmitt I. (2020). Unraveling the pharmacological potential of lichen extracts in the context of cancer and inflammation with a broad screening approach. Front. Pharmacol..

[81] Geng X., Zhang X., Zhou B., Zhang C., Tu J., Chen X., Wang J., Gao H., Qin G., Pan W. (2018). Usnic acid induces cycle arrest, apoptosis, and autophagy in gastric cancer cells in vitro and in vivo. Med. Sci. Monit..

[82] Eryilmaz I.E., Guney E.G., Egeli U., Yurdacan B., Cecener G., Tunca B. (2018). In vitro cytotoxic and antiproliferative effects of usnic acid on hormone-dependent breast and prostate cancer cells. J. Biochem. Mol. Toxicol..

[83] Backorova M., Backor M., Mikes J., Jendzelovsky R., Fedorocko P. (2011). Variable responses of different human cancer cells to the lichen compounds parietin, atranorin, usnic acid and gyrophoric acid. Toxicol. Vitr..

[84] Yu D.Y., Guo X.L., Gao H.Y., Cao H., Shi L.Y. (2020). Effects of usnic acid on proliferation and apoptosis of three cancer cell lines. J. Tianjin Norm. Univ..

[85] Sun Y., Wang H.J., Zhang W., Wu Y., Zhang Z.Q., Feng L., Wang L.Q., Wu Y.M. (2005). Preliminary study on the inhibition effect of usnic acid on proliferation prostate cancer PC-3M cells. Chin. J. Cancer Biother..

[86] Sun T.X., Li M.Y., Zhang Z.H., Wang J.Y., Xing Y., Ri M., Jin C.H., Xu G.H., Piao L.X., Jin H.L. (2021). Usnic acid suppresses cervical cancer cell proliferation by inhibiting PD-L1 expression and enhancing T-lymphocyte tumor-killing activity. Phytother. Res..

[87] Hao K.H., Han T., Hu P.B. (2016). Study on inhibitory effect and mechanism of usnic acid on H22 Tumor in mice. China Pharm..

[88] Song Y., Dai F., Zhai D., Dong Y., Zhang J., Lu B., Luo J., Liu M., Yi Z. (2012). Usnic acid inhibits breast tumor angiogenesis and growth by suppressing VEGFR2-mediated AKT and ERK1/2 signaling pathways. Angiogenesis.

[89] Kupchan S.M., Kopperman H.L. (1975). L-usnic acid: Tumor inhibitor isolated from lichens. Experientia.

[90] Jin J.Q., Ding D.N., Ouyang X.Y., Yan B.Q. (1996). Study on extraction and anticancer activity of usnic acid. Northwest Pharm. J..

[91] Millot M., Kaouadji M., Champavier Y., Gamond A.L., Simon A., Chulia A.J. (2013). Usnic acid derivatives from Leprocaulon microscopicum. Phytochem. Lett..

[92] Bazin M.A., Le Lamer A.C., Delcros J.G., Rouaud I., Uriac P., Boustie J., Corbel J.C., Tomasi S. (2008). Synthesis and cytotoxic activities of usnic acid derivatives. Bioorg. Med. Chem..

[93] Bezivin C., Tomasi S., Rouaud I., Delcros J.G., Boustie J. (2004). Cytotoxic activity of compounds from the lichen: *Cladonia Convoluta*. Planta Med..

[94] Takai M., Uehara Y., Beisler J.A. (1979). Usnic acid derivatives as potential antineoplastic agents. J. Med. Chem..

[95] Fernández-Moriano C., Divakar P.K., Crespo A., Gómez-Serranillos M.P. (2017). Protective effects of lichen metabolites evernic and usnic acids against redox impairment-mediated cytotoxicity in central nervous system-like cells. Food Chem. Toxicol..

[96] Erfani S., Valadbeigi T., Aboutaleb N., Karimi N., Moghimi A., Khaksari M. (2020). Usnic acid improves memory impairment after cerebral ischemia/reperfusion injuries by anti-neuroinflammatory, anti-oxidant, and anti-apoptotic properties. Iran. J. Basic Med. Sci..

[97] Odabasoglu F., Cakir A., Suleyman H., Aslan A., Bayir Y., Halici M., Kazaz C. (2006). Gastroprotective and antioxidant effects of usnic acid on indomethacin-induced gastric ulcer in rats. J. Ethnopharmacol..

[98] Odabasoglu F., Aslan A., Cakir A., Suleyman H., Karagoz Y., Halici M., Bayir Y. (2004). Comparison of antioxidant activity and phenolic content of three lichen species. Phytother. Res..

[99] Jin J.Q., Dong Y.L., He L.C. (2005). Experimental study on the effect of sodium usnic acid on skin wound healing. J. Chin. Med. Mater..

[100] Pagano C., Ceccarini M.R., Calarco P., Scuota S., Conte C., Primavilla S., Ricci M., Perioli L. (2019). Bioadhesive polymeric films based on usnic acid for burn wound treatment: Antibacterial and cytotoxicity studies. Colloids Surf. B.

[101] O’Leary R., Wood E.J., Guillou P.J. (2002). Pathological scarring: Strategic interventions. Eur. J. Surg..

[102] Wang P., Jiang L.Z., Xue B. (2016). Recombinant human endostatin reduces hypertrophic scar formation in rabbit ear model through down-regulation of VEGF and TIMP-1. Afr. Health Sci..

[103] Kwak D.H., Bae T.H., Kim W.S., Kim H.K. (2016). Anti-Vascular endothelial growth factor (Bevacizumab) therapy reduces hypertrophic scar formation in a rabbit ear wounding model. Arch. Plast. Surg..

[104] Zheng J., Song F., Lu S.L., Wang X.Q. (2014). Dynamic hypoxia in scar tissue during human hypertrophic scar progression. Dermatol. Surg..

[105] Song Y., Yu Z., Song B., Guo S., Lei L., Ma X., Su Y. (2018). Usnic acid inhibits hypertrophic scarring in a rabbit ear model by suppressing scar tissue angiogenesis. Biomed. Pharmacother..

[106] Koparal A.T. (2015). Anti-angiogenic and antiproliferative properties of the lichen substances (−)-usnic acid and vulpinic acid. Z. Naturforsch. C J. Biosci..

[107] Verotta L., Appendino G., Bombardelli E., Brun R. (2007). In vitro antimalarial activity of hyperforin, a prenylated acylphloroglucinol. A structure-activity study. Bioorganic Med. Chem. Lett..

[108] Bruno M., Trucchi B., Monti D., Romeo S., Kaiser M., Verotta L. (2013). Synthesis of a potent antimalarial agent through natural products conjugation. Chem. Med. Chem..

[109] Liver Doctor Editorial Department (2006). The mortality of drug induced liver disease ranked at the fifth in the world. Liver Doctor.

[110] Abo-Khatwa A.N., Al-Robai A.A., Al-Jawhari D.A. (1996). Lichen acids as uncouplers of oxidative phosphorylation of mouse-liver mitochondria. Nat. Toxins.

[111] Pramyothin P., Janthasoot W., Pongnimitprasert N., Phrukudom S., Ruangrungsi N. (2004). Hepatotoxic effect of (+)usnic acid from Usnea siamensis Wainio in rats, isolated rat hepatocytes and isolated rat liver mitochondria. J. Ethnopharmacol..

[112] Liu Q., Zhao X., Lu X., Fan X., Wang Y. (2012). Proteomic study on usnic-acid-induced hepatotoxicity in rats. J. Agric. Food Chem..

[113] Lu X., Zhao Q., Tian Y., Xiao S., Jin T., Fan X. (2011). A metabonomic characterization of (+)-usnic acid-induced liver injury by gas chromatography-mass spectrometry-based metabolic profiling of the plasma and liver in rat. Int. J. Toxicol..

[114] Han D., Matsumaru K., Rettori D., Kaplowitz N. (2004). Usnic acid-induced necrosis of cultured mouse hepatocytes: Inhibition of mitochondrial function and oxidative stress. Biochem. Pharmacol..

[115] Chen S., Zhang Z., Qing T., Ren Z., Yu D., Couch L., Ning B., Mei N., Shi L., Tolleson W.H. (2017). Activation of the Nrf2 signaling pathway in usnic acid-induced toxicity in HepG2 cells. Arch. Toxicol..

[116] Kwong S.P., Huang Z., Ji L., Wang C. (2021). PORIMIN: The key to (+)-Usnic acid-induced liver toxicity and oncotic cell death in normal human L02 liver cells. J. Ethnopharmacol..

[117] Shi Q., Greenhaw J., Salminen W.F. (2014). Inhibition of cytochrome P450s enhances (+)-usnic acid cytotoxicity in primary cultured rat hepatocytes. J. Appl. Toxicol..

[118] Emmerich R., Giez I., Lange O.L., Proksch P. (1993). Toxicity and antifeedant activity of lichen compounds against the polyphagous herbivorous insect Spodoptera littoralis. Phytochemistry.

[119] Cetin H., Tufan-Cetin O., Turk A.O., Tay T., Candan M., Yanikoglu A., Sumbul H. (2008). Insecticidal activity of major lichen compounds, (−)- and (+)-usnic acid, against the larvae of house mosquito. *Culex pipiens* L.. Parasitol. Res..

[120] Hesbacher S., Baur B., Baur A., Proksch P. (1995). Sequestration of lichen compounds by three species of terrestrial snails. J. Chem. Ecol..

[121] Roach J.A., Musser S.M., Morehouse K., Woo J.Y. (2006). Determination of usnic acid in lichen toxic to elk by liquid chromatography with ultraviolet and tandem mass spectrometry detection. J. Agric. Food Chem..

[122] Dailey R.N., Montgomery D.L., Ingram J.T., Siemion R., Vasquez M., Raisbeck M.F. (2008). Toxicity of the lichen secondary metabolite (+)-usnic acid in domestic sheep. Vet. Pathol..

[123] Long Y., Kirkpatrick C.R., Tai Z., Lin X. (1994). Report on the distribution, population, and ecology of the yunnan snub-nosed monkey (Rhinopithecus bieti). Primates.

[124] Grueter C.C., Li D., Ren B., Wei F., Xiang Z., van Schaik C.P. (2009). Fallback foods of temperate-living primates: A case study on snub-nosed monkeys. Am. J. Phys. Anthr..

[125] Huo S. (2005). Diet and Habitat Use of Rhinopithecus Bieti at Mt Longma, Yunnan, and Phylogeny of the Family Viverridae in China. Ph.D. Thesis.

[126] Xiang Z. (2005). The Ecology and Behavior of Black-and-White Snub-Nosed Monkeys (Rhinopithecus Bieti, Colobinae) at Xiaochangdu in Honglaxueshan National Nature Reserve, Tibet, China. Ph.D. Thesis.

[127] Li D., Ren B., He X., Hu G., Li B., Li M. (2011). Feeding habits of Yunnan snub-nosed monkey in Baima Snow Mountain Nature Reserve. Acta Theriol. Sin..

[128] Yu X.L. (2020). Study on the Differences of Intestinal Microbial Community Structure between Male and Female Yunnan Snub-Nosed Monkeys in Different Seasons. Master’s Thesis.

[129] Klein D.R. (1982). Fire, lichens, and caribou. J. Range Manag..

[130] Aagnes T., Mathiesen S. (1994). Food and snow intake, body mass and rumen function in reindeer fed lichen and subsequently starved for 4 days. Rangifer.

[131] Horand R. (1907). Mains de crocodile dermatose professionelle produite par le bois de chataignier. Gaz. Hop. Civ. Mil..

[132] Aalto-Korte K., Lauerma A., Alanko K. (2005). Occupational allergic contact dermatitis from lichens in present-day Finland. Contact Dermat..

[133] Mitchell J.C., Chan-Yeung M. (1974). Contact allergy from Frullania and respiratory allergy from Thuja. Can. Med. Assoc. J..

[134] Rademaker M. (2000). Allergy to lichen acids in a fragrance. Australas. J. Dermatol..

[135] Heine A., Tarnick M. (1987). Allergic contact eczema caused by usnic acid in deoderant sprays. Dermatol. Mon..

[136] Sheu M., Simpson E.L., Law S.V., Storrs F.J. (2006). Allergic contact dermatitis from a natural deodorant: A report of 4 cases associated with lichen acid mix allergy. J. Am. Acad. Dermatol..

[137] Hausen B.M., Emde L., Marks V. (1993). An investigation of the allergenic constituents of Cladonia stellaris (Opiz) Pous & Vezda (‘silver moss’, ‘reindeer moss’ or ‘reindeer lichen’). Contact Dermat..

[138] Ling G. (2018). Occupational airborne allergic contact dermatitis to usnic acid in an office-based dentis. J. Am. Acad. Dermatol..

[139] Krishna D.R., Ramana D.V., Mamidi N.V. (1995). In vitro protein binding and tissue distribution of D(+) usnic acid. Drug Metabol. Drug Interact..

[140] Foti R.S., Dickmann L.J., Davis J.A., Greene R.J., Hill J.J., Howard M.L., Pearson J.T., Rock D.A., Tay J.C., Wahlstrom J.L. (2008). Metabolism and related human risk factors for hepatic damage by usnic acid containing nutritional supplements. Xenobiotica.

[141] Hou L., Jin Y., Sun W., Guan S., Xu H., Wang Q., Zhang L., Du Y. (2019). Metabolites identification of (+)-usnic acid in vivo by ultra-high-performance liquid chromatography coupled with quadrupole time-of-flight mass spectrometry. Fitoterapia.

[142] Mitchell J.R., Jollow D.J., Potter W.Z., Davis D.C., Gillette J.R., Brodie B.B. (1973). Acetaminophen-induced hepatic necrosis. I. Role of drug metabolism. J. Pharmacol. Exp. Ther..

[143] Nakayama S., Atsumi R., Takakusa H., Kobayashi Y., Kurihara A., Nagai Y., Nakai D., Okazaki O. (2009). A zone classification system for risk assessment of idiosyncratic drug toxicity using daily dose and covalent binding. Drug Metab. Dispos..

[144] Piska K., Galanty A., Koczurkiewicz P., Zmudzki P., Potaczek J., Podolak I., Pekala E. (2018). Usnic acid reactive metabolites formation in human, rat, and mice microsomes. Implication for hepatotoxicity. Food Chem. Toxicol..

[145] Venkataramana D., Krishna D.R. (1993). Pharmacokinetics of d(+)-usnic acid in rabbits after intravenous administration. Eur. J. Drug Metab. Pharmacokinet..

[146] Krishna D.R., Venkataramana D. (1992). Pharmacokinetics of D(+)-usnic acid in rabbits after intravenous and oral administration. Drug Metab. Dispos..

[147] Wang H.X., Yang T., Cheng X.M., Kwong S.P., Liu C.H., An R., Li G.W., Wang X.H., Wang C.H. (2018). Simultaneous determination of usnic, diffractaic, evernic and barbatic acids in rat plasma by ultra-high-performance liquid chromatography-quadrupole exactive Orbitrap mass spectrometry and its application to pharmacokinetic studies. Biomed. Chromatogr..

[148] Fang W., Wang K.P., Han D.E. (2022). Preparation and in vivo pharmacokinetic study of usnic acid nanosuspension. Chin. Tradit. Pat. Med..

[149] Song T., Song D., Guan H.Y., Ding R., Zeng Z., Xu X.Y., Zhao Y. (2018). Study on pharmacokinetics and tissue distribution of usnic acid phospholipid complex in rats. Chin. Tradit. Herb. Drugs.

